# Hypophonia as only presenting symptom in myasthenia gravis – a diagnostic dilemma in poor countries: a case report

**DOI:** 10.1186/s13256-019-1970-6

**Published:** 2019-03-02

**Authors:** Avinash Chandra, Basant Pant

**Affiliations:** 1 Deptartment of Neurology, Annapurna Neurological Institute and Allied Sciences, Maitighar, Kathmandu, 44600 Nepal; 2 Deptartment of Neurosurgery, Annapurna Neurological Institute and Allied Sciences, Maitighar, Kathmandu, Nepal

**Keywords:** Autoimmune, Bulbar, Case report, Hypophonia, Myasthenia gravis

## Abstract

**Introduction:**

The autoimmune disease myasthenia gravis can mimic a variety of neurological disorders leading to a delay in diagnosis and treatment. In the older population, due to confusion with signs of the ageing process or comorbidities due to ageing, there are many underdiagnosed or misdiagnosed cases of myasthenia gravis. A majority of myasthenia gravis symptoms appear as ocular or motor symptoms and there are very few cases of bulbar symptoms. We present a case of myasthenia gravis with only hypophonia as a clinical feature.

**Case presentation:**

We present a case of a 51-year-old Madheshi woman whose only complaint was sudden onset of hypophonia which later showed a fluctuating nature throughout the daytime. There was only reduced pitch in her voice with no nasal tone or dysarthria (so-called dysphonia), which created a diagnostic dilemma. Later, a neurological examination and other relevant investigations suggested myasthenia gravis.

**Conclusions:**

Sudden onset and focal neurological deficit can raise the diagnostic dilemma of myasthenia gravis. Relevant laboratory tests and clinical examinations are important to diagnose this disease properly. In resources-deprived nations like Nepal, where several investigations are expensive and access to them is difficult, it becomes very difficult to achieve a solid diagnosis for rare presentations of diseases.

## Introduction

Myasthenia gravis (MG) is an autoimmune disease caused by a decrease of (AchR) and loss of postsynaptic receptors [1, 2]. A majority (> 50%) of patients with MG have ocular symptoms at the beginning of MG [3]. Only a minority of patients with this disease have bulbar symptoms, which can lead to dysarthria, dysphasia, or dysphonia [2–4]. For these reasons among many others, diagnosis of MG remains challenging [5]. Dysphonia as a primary clinical presentation of MG is particularly rare. A large case series consisting of only laryngeal features in MG (approximately 30 cases) had only one case with dysphonia [6].

## Case presentation

A 51-year-old Madheshi woman came to our neurology clinic with the chief complaint of sudden decrease in the tone and texture of her voice for the past 15 days. Her voice was very feeble but understandable and she noticed it was better by the time she got up from her bed only to worsen through the day to become nearly inaudible. She had noticed a slight change in her voice texture in the first several days which she had ignored in the beginning as it was not worth bothering about. She had no complaint of choking or coughing or aspiration or any throat discomfort. When asked for any other related and/or unrelated things she noticed in her habits, she complained about having constipation for many years otherwise she was apparently well. She had never visited hospital for any problem and no interventions had been done in the past. She denied smoking tobacco, drinking, or any other recreational drugs abuse. There was no one in her family or her parents’ family with any kind of known chronic disease. Her biological father had controlled hypertension with medication. Her psychosocial history was not significant. She came from a middle income family, and she had retired recently as an accountant for a small company. Her symptom progressively aggravated in later days to the extent that she not able to produce sound properly. It usually waned in the early morning or after enough voice rest only to wax throughout the day.

On physical examination, a systemic examination did not reveal any abnormality. On neurological examination, her muscle power was intact: 15/15 on Medical Research Council (MRC) scale. She had no imbalance and was able to tandem on walking. An examination of her gag reflex and other cranial nerves revealed no abnormal reflexes. Deep tendon reflexes were intact. Her speech articulation was intact and revealed no scanning of speech. Speech production was adequate and non-painful; her tone was non-nasal but the intensity was low and slow. Other neurological examinations also did not reveal any abnormality. Other systemic examinations were also non-significant.

Concerned with her problem, she had visited an ear, nose, and throat (ENT) department for her problem and was screened for possible laryngeal disorder for hypophonia. There was no obvious laryngeal pathology found and the treatment initiated by the ENT department had no satisfying outcome. She visited our neurology clinic. The differential diagnoses considered were Parkinson’s disease (PD) or a bulbar variant of motor neuron disease (MND), and MG. MRI scanning of her brain and screening of her whole spine appeared normal. Laboratory investigations including a hematologic panel, infectious disease screening panel, and myopathy panel involving creatinine kinase and creatine phosphokinase-N-acetyl cysteine (CPK-Nac), were all within normal range. She was suspected to have MG as other diseases were ruled out. A Quantitative Myasthenia Gravis (QMG) test was performed on our patient and her QMG score was 1 (for speech). She was given neostigmine challenge test with intravenously administered injection of 2.5 mg neostigmine and the result was positive after more than 30 minutes. There was significant improvement in her voice quality and she felt her fatigability improved as well. She gained a good quality of speech within an hour. QMG scoring was repeated, and she scored QMG score of 0. The longer duration of the medicine gave us enough time to assess the improvement. The laboratory test for the AchR antibody (AchR ab) is not available in Nepal. We had to send AchR ab to India, and the report received after waiting several weeks was positive. Quantitatively, it was significantly higher, 1.11 nmol/l than the normal range (0.0–0.4 nmol/l). She was later maintained on 60 mg of orally administered pyridostigmine with 12 hourly administrations and subsequently increasing to 6 hourly along with steroids.

Plain and contrast-enhanced computed tomography (CT) scans of her head, neck, and chest were done, and they revealed a mass in the anterior mediastinum: a well-defined, smooth outlined, approximately 4 × 2.5 × 2 cm, soft density nodule showing poor enhancement in the anterior mediastinum in the thymic space with maintained fat plane with the adjacent vessels and structure. Thymoma was suspected and she was sent to the surgical department for further surgical intervention.

She returned to our neurology clinic around 2 months after her first visit to us as a follow-up visit. She had undergone a successful surgery. She was doing well. Prednisolone was already tapered off. She was tapering down pyridostigmine as well, with a maintenance dosage of pyridostigmine 60 mg 12 hourly and planned to put off the medication. Her voice was clear and well maintained and she had no complaint of fatigue. She had no fresh complaint and was doing all her daily and professional activities very well.

## Discussion

MG presents mostly with fluctuating fatigue and weakness of a specific muscle group. Because of confusion with signs of the ageing process or comorbidities due to ageing, a large section of the older population are either underdiagnosed or misdiagnosed as MG [7]. Several laboratory investigations and clinical expertise are required for a proper diagnosis of MG. In the context of resource-deprived nations like Nepal, several of the investigations are not readily available. Lack of health care or subsidy adds further difficulties in getting investigations done due to extra expenses. This adds fuels to the diagnostic dilemma for health care professionals. MG is still mainly a clinically diagnosed disease characterized mostly by fluctuating symptoms. However, investigations are needed to cement the diagnosis. Investigations are laboratory testing, electrophysiological testing, and pharmacological testing. The pharmacological testing is done with edrophonium chloride or neostigmine or pyridostigmine. Electrophysiological testing such as electromyographic testing is done with repetitive nerve stimulation (RNS) and/or single-fiber electromyography (SFEMG); laboratory tests are mainly the serologic value of AchR or muscle-specific tyrosine kinase (MUSK) antibodies, and other tests such as CPK-Nac to rule out other possible causes.

The fatigability of the peripheral skeletal muscle is the hallmark of the disease and it can be absent in bulbar forms [8]. The bulbar form of MG would present as dysarthria, chewing fatigue, and/or dysphasia [9]. A case of a voice fatigue was also reported in one of the case reports [10]. However, in focal bulbar weakness, MND is suspected first [11]. Similarly, hypophonia without obvious dysarthria is more commonly seen in PD [12] with slow but progressive speech intensity decay [12]. As stated earlier, few case reports have been published regarding bulbar weakness like dysphonia [13, 14] in MG. They argued that there is palatal and/or laryngeal weakness in MG [15] and it can be a presenting symptom in late-onset MG [14]. We present a relatively rarer presentation of MG. Our case had a sudden onset and fluctuating hypophonia without obvious palatal weakness or nasal tone. This is different from the various cases that have been reported previously. Previous case reports discussed other bulbar features such as dysphasia, aspirations, vocal fatigability, or nasal tones [13, 14]. Our case had apparently none of these or any other problem other than hypophonia. For hypophonia, she had multiple other non-neurological consultations including ENT consultations without any improvement. Due to unavailability of electroglottography (EGG) and speech acoustics, she was evaluated by the ENT department for palatal weakness and they reported it to be as normal. Hence, without these standard facilities, we rely on physical examinations, clinical experience, and available laboratory tests to diagnose the disease. With the symptoms, we had a strong suspicion of MG. The QMG test is easy to administer and correlates well with activities of daily living [16]. SFEMG is more specific in MG [17] but technically more demanding and is also beyond our reach in Nepal, at present. Positive AchR ab confirms MG in a patient with appropriate symptoms and clinical findings [18, 19]. The laboratory report of our patient revealed high AchR (1.11 ng/ml). Of patients with thymoma, 20–25% have MG and 10–20% of patients with MG have thymoma [20, 21]. Patients with MG with thymoma are older and symptoms are more severe. Plain and enhanced CT scans of our patient’s chest and neck showed the presence of thymoma. Approximately 70% of cases of thymoma in patients with MG have titin and ryanodine receptor (RyR) antibodies. Unfortunately, again due to the limitation of our resources, a MUSK, RyR, or titin receptor antibodies assay could not be performed in our patient. Pyridostigmine is considered the first line of therapy. Our patient responded well to orally administered pyridostigmine and the drug treatment led to a rapid improvement in her speech quality and vocal symptoms. She was maintained on pyridostigmine and subsequently sent to the thoracic surgery department for surgical management of the thymoma. An informed written consent was obtained to publish this case report.

## Conclusion

The diagnosis for MG should not only rely on or wait for laboratory investigations. Even in focal bulbar weakness, like the hypophonia in our case, there should be a high index of suspicion to exclude MG. Early diagnosis and early necessary treatment from a multidisciplinary team enables improvement of MG and, in some cases, even total remission.

## Abbreviations

AchR: Acetylcholine receptor
AchR ab: Acetylcholine receptor antibody
CPK-Nac: Creatine phosphokinase-N-acetyl cysteine
CT: Computed tomography
EGG: Electroglottography
ENT: Ear, nose, and throat
MG: Myasthenia gravis
MND: Motor neuron disease
MRC: Medical Research Council
MUSK: Muscle-specific tyrosine kinase
PD: Parkinson’s disease
QMG: Quantitative Myasthenia Gravis
RNS: Repetitive nerve stimulation
RyR: Ryanodine receptor
SFEMG: Single-fiber electromyography
